# CKD Management in the Age of Telenephrology: An Observational Analysis of a Hybrid Telenephrology System within a Veteran's Affairs Medical Center

**DOI:** 10.34067/KID.0000000641

**Published:** 2024-11-22

**Authors:** Zachary Albert Scherzer, Brad C. Astor, Dyan Lesnik, Laura Maursetter

**Affiliations:** 1 University of Wisconsin School of Medicine and Public Health, Madison, Wisconsin; 2 William S. Middleton Memorial Veterans Hospital, Madison, Wisconsin; 3 The Department of Population Health Sciences, University of Wisconsin, Madison, Wisconsin

**Keywords:** CKD, chronic kidney disease, chronic renal insufficiency, kidney disease, nephrology, progression, progression of renal failure, renal function decline, renal progression

## Abstract

**Key Points:**

- Primary use of telenephrology in a hybrid system was associated with similar CKD progression outcomes as those seen primarily in-person.
- A hybrid system incorporating predominant use of telenephrology may be noninferior to standard in-person care with regard to multiple CKD outcomes.

**Background:**

Nephrology has seen an uptake in transition to remote care delivery. The effect of telenephrology care on CKD progression is not well defined.

**Methods:**

We analyzed data from patients naturally selected for telenephrology versus standard in-person visits. Patients were seen across 4230 visits over a 2-year period at a nephrology clinic within the Veterans Affairs (VA) health system. Baseline characteristics and health profile data were assessed on the basis of grouping of individuals to the telenephrology group (>50% virtual visits) or in-person group (≤50% virtual visits). The slope of eGFR change over time was estimated for each patient using a random effects regression model and compared across groups using weighted linear regression models.

**Results:**

A total of 1098 patients comprised the final analysis. The groups were similar across baseline demographics and health profiles, although more cardiovascular disease, congestive heart failure, and diabetes mellitus were present in the in-person group. There was no significant difference in eGFR decline between groups, although those in telenephrology group trended toward less steep decline compared with those seen predominately in-person (telenephrology slope versus in-person slope; difference=0.81 ml/min per 1.73 m^2^; 95% confidence interval, −0.447 to 2.08; *P* = 0.21). Those seen primarily in-person had a similar degree of proteinuria compared with those in telenephrology (*P* = 0.12). All-cause mortality and incidence of outpatient RRT initiation was similar. Telenephrology patients had an average of 1.3 fewer emergency department visits per individual compared with their in-person counterpart (2.17 versus 3.44, *P* < 0.001), as well as fewer hospital admissions (1.59 versus 2.08, *P* = 0.02). Those in the in-person group were more often prescribed sodium glucose cotransporter 2 inhibitors, statins, nonsteroidal anti-inflammatory drugs, and potassium supplements.

**Conclusions:**

Data from this observational study within a VA health care system suggest that medically complex patients with multimorbid CKD can expect a similar rate of eGFR decline when care is delivered through a hybrid system that includes a majority of telenephrology when compared with those managed in face-to-face visits. Further studies are needed to corroborate findings and ensure generalizability outside of this VA system.

## Visual Abstract

**Figure:**
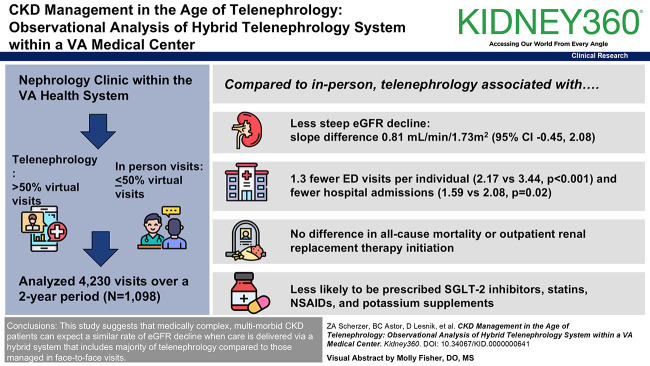

## Introduction

CKD is estimated to affect more than 13% of individuals worldwide, including 37 million people in the United States.^1,2^ The Veterans Affairs (VA) health system is particularly affected by a disproportionate prevalence of CKD within the veteran population. In the early 2000s, an evaluation of predialysis care within the VA revealed that more than one-third of veterans initiating dialysis did not receive preemptive nephrology care.^3^ The difficulty faced by the VA in delivering nephrology care is exacerbated by the regional distribution of veterans. Prior estimates have shown approximately 3.2 million VA enrollees, or 36% of total VA patients, reside in rural or remote parts of the country—almost twice the national average of US citizens.^4^ As such, the VA has sought to increase accessibility and timeliness of care delivery through establishing a robust system of telehealth.

Telehealth, as defined by the VA, is the “use [of] health informatics, disease management, and telecommunication technologies to target care and case management to improve access to care [and] the health of Veterans.^5^” When considering delivering care by this method, it is important to assess efficacy and outcomes of telemedicine in caring for patients with CKD compared with the usual (in-person) standard of care. Given that the VA has been a leader in implementing telehealth, many of the existing data involves VA patients. A study conducted by Tan *et al.*^6^ compared clinical outcomes between CKD patients from the geographically remote Hudson Valley VA Medical Center using telenephrology services and those receiving usual standard of in-person CKD care at the Bronx VA Medical Center in New York. Overall, there was no difference over 2 years of follow-up between the remote Hudson Valley telemedicine patients and their in-person counterparts for composite end point of death, ESKD, or doubling of serum creatinine (SCr). Furthermore, a retrospective and descriptive study evaluating the effect of a telenephrology clinic intervention in patients with CKD within the Miami VA Health Care System showed a statistically significant reduction in systolic BP while renal function (based upon SCr) was stabilized in patients receiving virtual care throughout the study.^7^ Finally, a randomized controlled trial completed by Ishani *et al.* ^8^ at the Minneapolis VA Health Care System showed noninferiority of an interprofessional telehealth delivery approach in patients with CKD compared with usual standard care with regard to a composite outcome of death, hospitalization, emergency department (ED) visits, or admission to skilled nursing facilities.

These studies have shown that telehealth can be delivered to patients with CKD with comparable outcomes to standard visits. In addition, telehealth has been able to enhance access to specialized care for those in rural areas and for those with limited mobility/transportation barriers. Furthermore, it has been fiscally cost-saving with reduced travel expense and reduced clinic space requirements, as well as improved patient visit adherence (decreased no-show rate).^6,9^ There are important limitations and barriers involved in this method of health care delivery that need mention when interpreting results: (1) the inability to perform an extensive physical examination, (2) factors that affect the ability to have the necessary technology or internet connections because of location or socioeconomic barriers may influence the population that can access telehealth, and (3) unique challenges faced by those with hearing/visual impairments.

Studies of telenephrology care have shown feasibility and effectiveness in CKD care. Several questions remain: Which patients are appropriate for telehealth visits? What are the implications of primarily telenephrology care on CKD management and progression? To date, there has been very little direction into optimal patient selection for the delivery of telenephrology care and investigation into the rate of CKD progression when using telehealth modalities. This study sought to review differences in patient populations that were provided and/or self-selected to telenephrology modalities in one VA nephrology clinic. In addition, the primary outcome sought to identify differences in the rate of eGFR decline between groups. Additional factors were reviewed to determine whether there were downstream consequences of the care delivery, such as increased hospitalizations, emergency room visits, or particular medication prescriptions.

## Methods

In this retrospective, observational quality improvement analysis, data from a centralized VA database were obtained from a nephrology clinic associated with the William S. Middleton Memorial Veteran's Hospital in Madison, WI. Within this clinic, a group of five attending nephrologists and six fellow physicians delivered nephrology care through standard in-person visits and through telenephrology.

### Study Population

Patients seen at the Madison VA were included in the study unless they were younger than 18 years, dialysis-dependent at baseline, or had fewer than five SCr measurements during the study period (to ensure a trend was achieved). The baseline data collection period spanned approximately 24 months (2021–2023) and a total of 4230 visits (Table 1).

**Table 1:** Baseline demographic and health profile data

Characteristic	In-Person	Telenephrology	*P* Value
No. of patients, *n*	882	216	
Average no. of telenephrology visits (SD)	0.59 (0.96)	2.76 (1.48)	<0.001^a^
Average no. of in-person visits (SD)	2.77 (1.86)	0.80 (0.87)	<0.001^a^
Average total visits (SD)	3.36 (2.37)	3.56 (2.09)	0.24
Average age (SD)	72.9 (10.7)	72.4 (10.8)	—
**Self-identified sex, %**			
Male	96.6	94.4	—
Female	3.4	5.6	—
**Self-identified race/ethnicity, %**			
Black	5.3	8.8	0.08
Other	2.6	4.2	—
White	92.1	87	—
**Locale, %**			
Urban	42.9	41.2	—
Rural	55.4	56	0.65
Homeless	1.7	2.8	—
**State, %**			
Wisconsin	78.1	68.5	—
Illinois	17.9	27.8	0.21
Other	4	3.7	—
**Marital status, %**			
Married	58.5	53.2	0.21
**Smoking status, %**			
Smoker (current/former)	33.6	34.3	0.9
**BMI, %**			
BMI >30	52.2	51.4	0.19
Hemoglobin A1c (average), %	6.79	6.61	0.09
CVD, %	21.4	15.7	0.02^a^
PVD, %	6.8	6	0.09
CHF, %	16.6	14.8	0.02^a^
COPD, %	12.6	12	0.99
DM, %	39.3	31	0.005^a^
Malignancy, %	23.4	19.4	0.4

BMI, body mass index; CHF, congestive heart failure; COPD, chronic obstructive pulmonary disease; CVD, cardiovascular disease; DM, diabetes mellitus; PVD, peripheral vascular disease.

a Statistically significant.

### Exposure

Standard in-person visits were conducted at a clinic within the William S. Middleton Memorial Veteran's Hospital. Visits categorized as telenephrology were conducted either through provider video call into a satellite VA clinic where a patient was roomed and vitals were obtained by a medical assistant or directly to a patient's home through personal computer (provided by the VA in instances of financial need). Visit modality for each appointment was determined by the individual providers on the basis of clinical context and without any formal protocol or education for patient-specific modality selection. Patient preference of modality was considered when provider selection was equivocal. Visit modality was also fluid throughout the study period for each individual patient, with the ability to transition from in-person visits to telenephrology and *vice versa* at subsequent visits. Patients were placed into groups on the basis of individual visit modality distribution across the study period (>50% telenephrology visits or ≤50% telenephrology visits).

### Covariates

Baseline demographic data included age (at outset of study period), self-identified sex, self-identified race/ethnicity, locale (rural versus urban, based upon zip code), homeless status, state of residence, marital status, smoking status (current or former versus never), body mass index, and baseline hemoglobin A1c. Incidence of common comorbid conditions was also collected through associated International Classification of Disease-10 codes from the electronic medical record, including cardiovascular disease (CVD), peripheral vascular disease, congestive heart failure (CHF), chronic obstructive pulmonary disease, diabetes mellitus (DM), and any malignancy.

### Outcomes

The primary outcome was the change in eGFR over the study period. Baseline SCr was identified at the start of the study period, and all additional SCr measurements across the 2-year study period were collected. SCr values used in the study analysis included any value collected during the study across all clinical encounters, which included those obtained before any nephrology visit. Secondary outcomes included total ED visits, hospital admissions (defined by ≥1 night in hospital), incidence of initiation of outpatient dialysis, palliative care consults, and all-cause mortality. Finally, frequency of commonly prescribed classes of medications, as denoted on patients’ medication list at any point during the study period, were assessed (Table 2).

**Table 2:** Frequency of commonly prescribed medications taken by patients

Medication	In-Person (*n*=882)	Telenephrology (*n*=216)	*P* Value
ACEi, %	50.68	46.76	0.30
ARB, %	27.10	22.69	0.19
MRA, %	21.66	16.67	0.11
SGLT2i, %	29.02	18.06	0.001^a^
Loop, %	45.01	48.61	0.34
Thiazide, %	24.83	23.61	0.71
BB, %	66.10	62.50	0.32
CCB, %	54.54	52.31	0.56
Statin, %	74.49	63.43	0.001^a^
Insulin, %	32.09	29.17	0.41
NSAID, %	7.94	3.24	0.02^a^
Potassium, %	5.22	1.39	0.02^a^
Bicarb, %	0.57	0.46	0.85

ACEi, angiotensin-converting enzyme inhibitor; ARB, angiotensin II receptor blocker; BB, *β* blocker; Bicarb, sodium bicarbonate; CCB, calcium channel blocker; Loop, loop diuretic; MRA, mineralocorticoid receptor antagonist; NSAID, nonsteroidal anti-inflammatory drug; SGLT2i, sodium glucose cotransporter 2 inhibitor; Thiazide, thiazide diuretic.

a Statistically significant.

### Statistical Analysis

GFR was estimated = using SCr in the 2021 CKD Epidemiology Collaboration equation.^10^ Change in eGFR was estimated for each patient using all estimates in a random effects regression model. Slopes estimated from these models were then compared across groups using linear regression models weighted to account for the variance of each patient's estimate. Similar analyses were performed for proteinuria. Proportions of patients with each secondary outcome were compared across visit groups by chi-square tests.

### Ethical Approval

Given the quality improvement/assurance nature of this project, formal Institutional Review Board review was not required pursuant to code 45 of federal regulations 46.102(d).

## Results

### Baseline Statistics

An initial population of 1231 patients were analyzed, with those having <5 eGFR measurements (*n*=133) removed from the final study analysis. A total of 1098 patients comprised the final study sample. The average number of visits per patient (any visit type) during the study period was comparable between the two groups (telenephrology 3.56 [2.09] versus in-person 3.36 [2.37], *P* = 0.24). There was a significant difference between the number of visits attended by the patients for their assigned group, confirming the distribution was different (Table 1). The two groups were similar across baseline demographic characteristics, although more CVD, CHF, and DM were present in the in-person group (Table 1). The average age at study outset was 72.8±10.7 years. Most patients identified as White (91.1%) and male sex (96.2%). Approximately two-thirds of patients were residents of Wisconsin, with no difference in locale (rural versus urban). A total of 33,390 eGFR measurements were obtained during the study period, with the average number of measurements per patient in each of the two groups being overall similar (Table 3). Baseline average eGFR of the total patient population was 48.27 ml/min per 1.73 m^2^±21.6 with no significant difference between the two groups. Average baseline proteinuria was comparable between groups (in-person=1089 mg/g and telenephrology=913 mg/g; *P* = 0.52).

**Table 3:** Baseline eGFR and UPC and change in eGFR and UPC per year of telenephrology compared to in-person visits

Measurement	In-Person	Telenephrology	*P* Value
Baseline eGFR	48.25 (22.0)	48.35 (20.2)	0.88
eGFR change, slope/yr	−0.23 (6.26)	0.92 (7.96)	0.41
eGFR change, %/yr	1.32 (25.9)	5.60 (44.1)	0.06
No. of creatinine values, average/patient	31.19 (28.51)	27.22 (27.55)	0.52
Baseline UPC, mg/g	1089 (1967)	913 (1547)	0.52
Slope, mg/g per year	−0.9 (311.5)	6.1 (217.8)	0.12
No. of UPC measurements, median (IQR)	5 (3–7)	4 (2–6)	—

eGFR, eGFR (ml/min per 1.74 m^2^); IQR, interquartile range; UPC, urine protein:creatine.

### Primary Outcome

**Figure 1:**
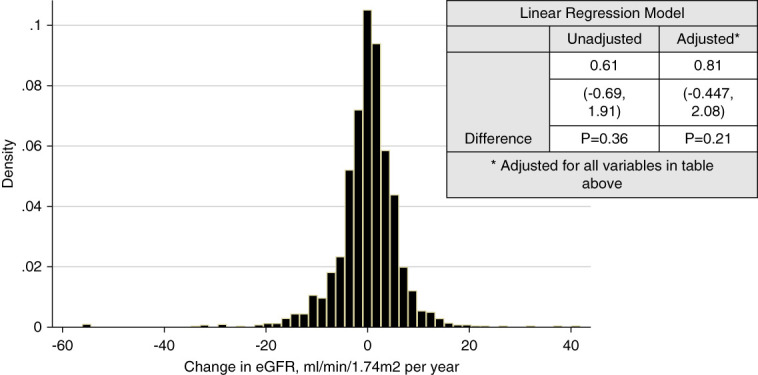
Weighted linear regression analysis of eGFR trends of telenephrology compared with in-person visits.

The Δ eGFR (slope/yr) during the study period was −0.23 ml/min per 1.73 m^2^ for those seen predominately in-person and 0.92 ml/min per 1.73 m^2^ (*P* = 0.41) for those seen primarily over telenephrology. When adjusting for all covariates, those in the telenephrology group trended toward greater eGFR compared with those seen in-person (0.81; [−0.447 to 2.08], *P* = 0.21) (Figure 1). A similar result of no difference in GFR decline was observed in a subsequent analysis comparing percent change in eGFR/yr between the in-person and telenephrology groups, respectively (1.32% versus 5.60%, *P* = 0.06).

### Secondary Outcomes

There was no significant difference in change in proteinuria status between in-person (−0.9 mg/g per year) and telenephrology groups (6.1 mg/g per year; *P* = 0.12). All-cause mortality and initiation of outpatient RRT was overall similar between groups (Table 4). Patients in the telenephrology group had an average of 1.3 fewer ED visits per individual compared with their in-person counterpart (2.17 versus 3.44, *P* < 0.001). Those in the telenephrology group also tended to have fewer hospital admissions (1.59 versus 2.08, *P* = 0.023). On average, palliative care was consulted for 15% of the total patient population, with similar frequency of consultation between groups (Table 4). The number of individuals taking commonly prescribed medications was overall similar between groups (Table 2). Significantly more patients in the in-person group were prescribed sodium glucose cotransporter 2 inhibitors (SGLT2i) (*P* = 0.001), statin medications (*P* = 0.001), as well as nonsteroidal anti-inflammatory drug (*P* = 0.02) and oral potassium supplements (*P* = 0.02).

**Table 4:** Secondary outcome data

Outcome	In-Person	Telenephrology	Average	Difference	*P* Value
Mortality, total no.	171	43	—	—	0.86
Dialysis initiation, %	6.60	5.10	—	—	0.42
ED visits, per patient	3.44	2.17	3.19	−1.27 (−1.96 to −0.59)	0.00^a^
Hospital admissions, per patient	2.08	1.59	1.98	−0.49 (−0.91 to −0.07)	0.02
Palliative consults, %	0.16	0.12	0.15	—	0.12

ED, emergency department.

a Statistically significant.

## Discussion

As patient care increasingly shifts toward a value-based model of health care delivery, propelled by the pandemic, it is essential to focus on disease-related outcomes to determine the standard of care for these modalities. The utility and effectiveness of telemedicine within the field of nephrology requires investigation to provide confidence in the value of this method of health care delivery. In this single-center, observational quality-improvement study comparing telenephrology with in-person, standard care management of CKD, no significant difference in CKD progression was noted between the two groups. Although not statistically significant, there was a trend toward improved eGFR in the telenephrology group, while those who received predominantly in-person care were noted to have a slight decline in eGFR during the study period.

To interpret these data, it is important to note differences between the groups. In the clinic platform studied, most patients received a mix of in-person and telenephrology care, with selection of modality based on provider selection, as well as patient preference. In the final analysis, patients in both groups had similar total number of visits with confirmation of a significant difference between groups in the method of care delivery. As expected, there were more comorbidities in the in-person group, with significantly more DM, CHF, CVD; however, the overall comparable health profiles, including similar metrics such as body mass index, A1c, and degree of baseline proteinuria, of the two groups, in addition to the comparable CKD progression outcomes, suggest that medically complex patients with multimorbid CKD do not have factors that cause faster rates of progression when managed through telenephrology.

A common concern associated with telemedicine is that aspects of care may be overlooked or missed given its remote nature. Telenephrology patients were seen in the ED fewer times than in-person counterparts and had fewer hospital admissions—two downstream clinical events that can commonly be seen as either direct or indirect results of missed or insufficient care (Table 4). Additional outcomes were similar between groups, suggesting that those managed primarily through telenephrology do not have heightened mortality or progression to dialysis dependence compared with their in-person counterparts, keeping in mind they did have less comorbidities (Table 4). Although this study did not expressly evaluate BP control between groups, results from a prior study conducted at the same VA medical center demonstrated that an interdisciplinary team comprised of a pharmacist and nephrologist using a virtual care model is an effective method for managing difficult-to-control hypertension.^11^ Similar BP stabilization (to the study of Ladino *et al.*) in difficult-to-control hypertensive patients with CKD was seen with use of an interdisciplinary telehealth team (pharmacist+nephrologist) at the same center.^11^

It is reassuring to see similar prescribing patterns of commonly indicated medications between the two groups (Table 2). Although this could be attributed to the similar health profile of patients in the two groups, it supports that important medications for management of CKD are not missed with telenephrology care. There were a few exceptions; importantly, the finding that SGLT2i and statins were more frequently prescribed to those in the in-person group is concerning. Specifically regarding SGLT2i, both groups were found to have comparable baseline proteinuria at a degree for which SGLT2i initiation would be indicated. Despite this, only 29% and 18% of in-person and telenephrology patients, respectively, were prescribed this important class of medication. On the basis of this prescription pattern, this could explain why there was nonsignificant improvement in proteinuria for the in-person group as compared with the telenephrology group. While this difference could be explained by a greater degree of comorbid CVD, CHF, and DM in the in-person group, which has triggered these prescriptions, it still highlights a clear need for improved prescriber pattern for this class of medication across both care modalities and especially in telenephrology. Nonsteroidal anti-inflammatory drugs were found to be prescribed twice as often to those seen in-person compared with telenephrology, while potassium supplements were prescribed almost four times as frequently to this group compared with those seen through telenephrology. While the underlying difference in potassium supplementation is unclear, one reason for this difference may include the greater proportion of CHF in this group and resultant increased diuretic use that may have led to associated hypokalemia requiring supplementation. Although not entirely clear, increased nonsteroidal anti-inflammatory disease use in the in-person group could signal the presence of more polypharmacy in this group or coincide with selection of patients needing enhanced education on renoprotective strategies into the in-person modality.

The findings in this study correlate with that of prior data within the VA health system. In a randomized, controlled trial at the Minneapolis VA, Ishani *et al.*^8^ demonstrated not only feasibility of a telehealth system of CKD management, but also no significant difference in composite outcome of death, hospitalization, ED visits, or admission to skilled nursing facilities compared with usual care. Similarly, at the Hudson Valley VA medical center, Tan *et al.*^6^ found no significant difference between in-person and telenephrology groups during a 2-year study period with composite end outcome of death, ESKD, or doubling of SCr (*P* = 0.96).

Our study did not focus on measures of patient or provider experience; however, this remains an important consideration when weighing the effect of telenephrology. Similar to the Tan *et al.* study,^6^ however, an analysis of clinic attendance based upon modality during our selected study period did show reduced no show visits in the telenephrology group as opposed to the in-person group (4.0% versus 6.1%, *P* = 0.03).

To our knowledge, there are few studies that have investigated effect of telenephrology on rate of CKD progression compared with in-person care. This study includes a large number of patients and associated clinical visits with outcomes that provide reassurance that remote care can be provided in comparative fashion to in-person care. Despite these findings, there are multiple limitations to this study. First, owing to institutional policies on data collection in quality-improvement studies, the time frame for the data analyzed in this study did partially overlap with the coronavirus disease 2019 pandemic, a factor that did likely influence care modality selection, with more telenephrology visits selected. Most individuals received care through both telenephrology and in-person throughout the study period, with grouping of individuals based on the most frequent modality of care received. Given the mix of care modalities, without purely one type of care received during the entirety of the study period, it is somewhat difficult to assess the effect of either care modality independently. Furthermore, while the predominantly White men demographic pattern of patients analyzed in this study is representative of the local and regional veteran population, racial differences in uptake of telemedicine and associated outcomes with this care platform have previously been established.^12^ Difference in socioeconomic factors could serve as a potential confounders for better outcomes and decreased incidence of comorbidities (CVD, CHF, DM) that may be more commonly seen in economically disadvantaged populations. Unfortunately, these factors were not available to be collected in this population to compare between groups. That said, the VA does have resources available to provide telehealth equipment, including devices and internet, to those who may not otherwise have access to telehealth. This makes the financial factors less of an issue in this study. Furthermore, provider selection to care modality may have also been influenced or biased by these factors. For these reasons, it may be difficult to generalize findings from our study, certainly to populations outside this VA system.

Overall, this observational study within a VA health system demonstrates that the predominant use of telenephrology based on provider discretion and clinical context in the management of CKD may be noninferior to majority in-person care, supporting possible broader utilization of this care modality. Further study is needed, however, on multiple fronts, including in alternative populations and health care subsets, to ensure generalizability and assist in informing, enhancing, and streamlining individual patient selection to maximize utility of telenephrology care.

## Supplementary Material

## Data Availability

All data are included in the manuscript and/or supporting information.
