# Supplementary material for: CKD Management in the Age of Telenephrology: An Observational Analysis of a Hybrid Telenephrology System within a Veteran's Affairs Medical Center
Source: Kidney360. 2024 Nov 22;6(1):69–75. doi: 10.34067/KID.0000000641 (PMC11793171; doi:10.34067/KID.0000000641)
Supplement: Supplementary file 1 [file kidney360-6-069-s001.pdf]

ASN American Society of Nephrology · JASN Journal of the American Society of Nephrology · KIDNEY360 Accessing Our World From Every Angle · CJASN Clinical Journal of the American Society of Nephrology

ASN Journal Disclosure Form

As per ASN journal policy, I have disclosed any financial relationships or commitments I have held in the past 36 months as included below. I have listed my Current Employer below to indicate there is a relationship requiring disclosure. If no relationship exists, my Current Employer is not listed.

B. Astor reports the following:
Employer: University of Wisconsin School of Medicine and Public Health; Consultancy: Vasc-Alert; and Other Interests or Relationships: Consultancy for Dialysis Vascular Access Consortium.

I understand that the information above will be published within the journal article, if accepted, and that failure to comply and/or to accurately and completely report the potential financial conflicts of interest could lead to the following: 1) Prior to publication, article rejection, or 2) Post-publication, sanctions ranging from, but not limited to, issuing a correction, reporting the inaccurate information to the authors' institution, banning authors from submitting work to ASN journals for varying lengths of time, and/or retraction of the published work.

Name: Brad C. Astor
Manuscript ID: Disclosure Form for K360-2024-000487R1
Manuscript Title: CKD Management in the Age of Telenephrology: An Observational Analysis of a Hybrid Telenephrology System
Date of Completion: September 16, 2024
Disclosure Updated Date: August 26, 2024

ASN
American Society of Nephrology
JASN
Journal of the American Society of Nephrology
KIDNEY360
Accessing Our World From Every Angle
CJASN
Clinical Journal of the American Society of Nephrology

ASN Journal Disclosure Form

As per ASN journal policy, I have disclosed any financial relationships or commitments I have held in the past 36 months as included below. I have listed my Current Employer below to indicate there is a relationship requiring disclosure. If no relationship exists, my Current Employer is not listed.

D. Lesnik reports the following:
Employer: Department of Veterans Affairs, Veterans Health Administration

I understand that the information above will be published within the journal article, if accepted, and that failure to comply and/or to accurately and completely report the potential financial conflicts of interest could lead to the following: 1) Prior to publication, article rejection, or 2) Post-publication, sanctions ranging from, but not limited to, issuing a correction, reporting the inaccurate information to the authors' institution, banning authors from submitting work to ASN journals for varying lengths of time, and/or retraction of the published work.

Name: Dyan M. Lesnik
Manuscript ID: K360-2024-000487R1
Manuscript Title: CKD Management in the Age of Telenephrology: An Observational Analysis of a Hybrid Telenephrology System
Date of Completion: September 17, 2024
Disclosure Updated Date: September 17, 2024

ASN American Society of Nephrology | JASN Journal of the American Society of Nephrology | KIDNEY360 Accessing Our World From Every Angle | CJASN Clinical Journal of the American Society of Nephrology

ASN Journal Disclosure Form

As per ASN journal policy, I have disclosed any financial relationships or commitments I have held in the past 36 months as included below. I have listed my Current Employer below to indicate there is a relationship requiring disclosure. If no relationship exists, my Current Employer is not listed.

L. Maursetter reports the following:
Employer: University of Wisconsin School of Medicine & Public Health; William S. Middleton Veterans Hospital; and Honoraria: ASN for BRCU; Vizient for a Mock Pharmacy and Therapeutics conference.

I understand that the information above will be published within the journal article, if accepted, and that failure to comply and/or to accurately and completely report the potential financial conflicts of interest could lead to the following: 1) Prior to publication, article rejection, or 2) Post-publication, sanctions ranging from, but not limited to, issuing a correction, reporting the inaccurate information to the authors' institution, banning authors from submitting work to ASN journals for varying lengths of time, and/or retraction of the published work.

Name: Laura J. Maursetter
Manuscript ID: K360-2024-000487R1
Manuscript Title: CKD Management in the Age of Telenephrology: An Observational Analysis of a Hybrid Telenephrology System
Date of Completion: October 15, 2024
Disclosure Updated Date: September 16, 2024

ASN American Society of Nephrology | JASN Journal of the American Society of Nephrology | KIDNEY360 Accessing Our World From Every Angle | CJASN Clinical Journal of the American Society of Nephrology

ASN Journal Disclosure Form

As per ASN journal policy, I have disclosed any financial relationships or commitments I have held in the past 36 months as included below. I have listed my Current Employer below to indicate there is a relationship requiring disclosure. If no relationship exists, my Current Employer is not listed.

Z. Scherzer reports the following:
Employer: University of Wisconsin Hospitals and Clinics; Agrace Hospice and Palliative Care

I understand that the information above will be published within the journal article, if accepted, and that failure to comply and/or to accurately and completely report the potential financial conflicts of interest could lead to the following: 1) Prior to publication, article rejection, or 2) Post-publication, sanctions ranging from, but not limited to, issuing a correction, reporting the inaccurate information to the authors' institution, banning authors from submitting work to ASN journals for varying lengths of time, and/or retraction of the published work.

Name: Zachary A. Scherzer
Manuscript ID: K360-2024-000487R1
Manuscript Title: CKD Management in the Age of Telenephrology: An Observational Analysis of a Hybrid Telenephrology System
Date of Completion: September 18, 2024
Disclosure Updated Date: September 18, 2024
